# Exploring potential pharmacological mechanisms of *Yiqi Tuomin* Decoction in the treatment of allergic rhinitis utilizing network pharmacology prediction and molecular docking-based strategies: experimental research

**DOI:** 10.1097/MS9.0000000000000804

**Published:** 2023-05-15

**Authors:** Weixin Zhang, Qing Zhou, Xiaoning Chen, Jingjing Zhao, Jun Shi, Li Chen

**Affiliations:** Affiliated Hospital of Nanjing University of Chinese Medicine, Nanjing City, People’s Republic of China

**Keywords:** allergic rhinitis, molecular docking, network pharmacology

## Abstract

*Yiqi Tuomin* Decoction (YTD), which originated from the theory of lung deficiency and cold in Chinese medicine, is a common Chinese herbal formula used against allergic rhinitis (AR). In our otolaryngology department, this prescription has been used to treat so many AR patients with lung-deficiency-related colds for nearly 30 years. However, the mechanism of its ingredient–target is still unclear. Based on our early experiments and clinical case studies, in this paper, we explore the mechanism of YTD systematically against AR using bioinformatic methods of network pharmacology and molecular docking.

**Methods:**

The Traditional Chinese Medicine Systems Pharmacology (TCMSP) database was used to screen the active ingredients and targets of YTD. The AR-related targets were retrieved from OMIM, GeneCards, TTD, DisGeNET, DrugBank databases, and PharmGKB. The Venn database was used to screen the potential core targets. After that, the STRING database was used to construct the protein–protein interaction (PPI) of the core targets and then visualize it by Cytoscape. The Gene Ontology (GO)-enriched processes and Kyoto Encyclopedia of Genes and Genomes (KEGG) pathways of the core targets were analyzed by the KOBAS-I database and Sangerbox. Molecular docking was used to assess interactions between potential targets and active ingredients.

**Results:**

A total of 169 active ingredients and 238 targets of YTD were predicted. YTD shared 115 common targets with AR from the Venn database. The GO-enriched processes and KEGG pathways indicate that genes involved in inflammation and oxidative stress, accompanying the MAPK signaling pathway, Th17 cell differentiation, IL-17 signaling pathway, and Th1 and Th2 cell differentiation, may play a mediated effect in YTD. The docking results showed good binding ability between the active ingredients and the selected targets.

**Conclusions:**

Our study systematically indicated the underlying mechanism of YTD against AR from the perspective of bioinformatics. By studying the active ingredients of YTD, we obtained molecular mechanisms and established a reliable method and molecular theoretical basis for the sensible development of Chinese medicine in the treatment of AR.

## Introduction

Highlights

- Investigating the anti-allergic rhinitis (AR) pharmacological mechanism of *Yiqi Tuomin* Decoction (YTD) by network pharmacology and molecular docking.
- Key anti-AR targets include JUN, STAT3, and IL6.
- Wogonin, 7-methoxy-2-methyl isoflavone, formononetin, and beta-sitosterol might be the core bioactive ingredients from YTD that can treat AR.

Allergic rhinitis (AR) is a common condition^1^. As early as 2001, the World Health Organization (WHO) released a report on AR and its impact on asthma^2^. The epidemiological studies suggested that 20–30% of adults and ~40% of children were affected^3^. In industrialized areas, allergic sensitization and airway responsiveness to allergens are initially presented due to the increasing combustion of fossil fuels^4^. The symptoms of AR may be aggravated due to subsequent airway responsiveness to environmental allergens^5^. AR is a chronic inflammatory disease of the airways that is caused by an allergic response mediated by immunoglobulin E and Th2 lymphocytes in the nasal mucosa. AR itself is not a serious disease, but it is a substantial socioeconomic burden. The induced symptoms, such as headache, nasal congestion, sneezing, reduced operating efficiency, and frequent complications with allergic asthma, can worsen the illness year by year. This can eventually lead to perennial asthma, emphysema, and cor pulmonale^6^, which can have a significant negative impact on patients. In the process of treating AR, allergen immunotherapy (AIT) is an acknowledged and irreplaceable clinical choice for the treatment of AR^7,8^. However, currently, the authorized biomarkers of AIT that predicted success have not been presented. Some patients with AR still experience exacerbations associated with allergen exposure despite being under drug treatment. Therefore, the easier the new drugs and treatments available, the better for AR patients.

Traditional Chinese medicine (TCM) has existed in China for thousands of years as a traditional medicine. Different from orthodox medicine, aiming to examine the pathogenic mechanisms^9,10^, TCM is known for possessing ‘multi-ingredient, multi-target and multi-pathway’ characteristics. TCM has a long history in treating AR. AR is classified as ‘Bi Qiu’ in TCM^11^, which first appeared in *Huangdi Neijing*. TCM adopts several approaches in the treatment of AR, such as oral or external applications of Chinese medicine and acupuncture^11^. *Yiqi Tuomin* Decoction (YTD), which originated from the theory of lung deficiency and cold of Chinese medicine, comprises 12 Chinese herbal ingredients and treats different patients with different ingredient ratios. Clinical cases have shown that YTD has therapeutic effects on AR by improving the deficiency of Lung Qi, consolidating the defensive Qi, reducing airway inflammation, and regulating mucus secretion. However, the underlying pharmacological mechanism is not fully clear.

Network pharmacology belongs to the category of systems biology and multidirectional pharmacology^12^ and is an emerging avenue for drug research and development for TCM herbs or herbal formulas^13^. Therefore, firstly, we screened the active ingredients and targets of YTD. Next, we identified the common targets of AR from databases. Finally, we understood the underlying mechanism and the key shared targets. Secondly, we constructed the core PPI network, categorized the PPI network to investigate the key pharmacological mechanisms of YTD against AR through cluster analysis, and investigated the signaling pathways of biological function by performing the enrichment analysis of Gene Ontology (GO) and Kyoto Encyclopedia of Genes and Genomes (KEGG) pathways. Finally, the molecular docking method was taken to verify the interaction between the screened bioactive ingredients and the key common targets.

**Figure 1:**
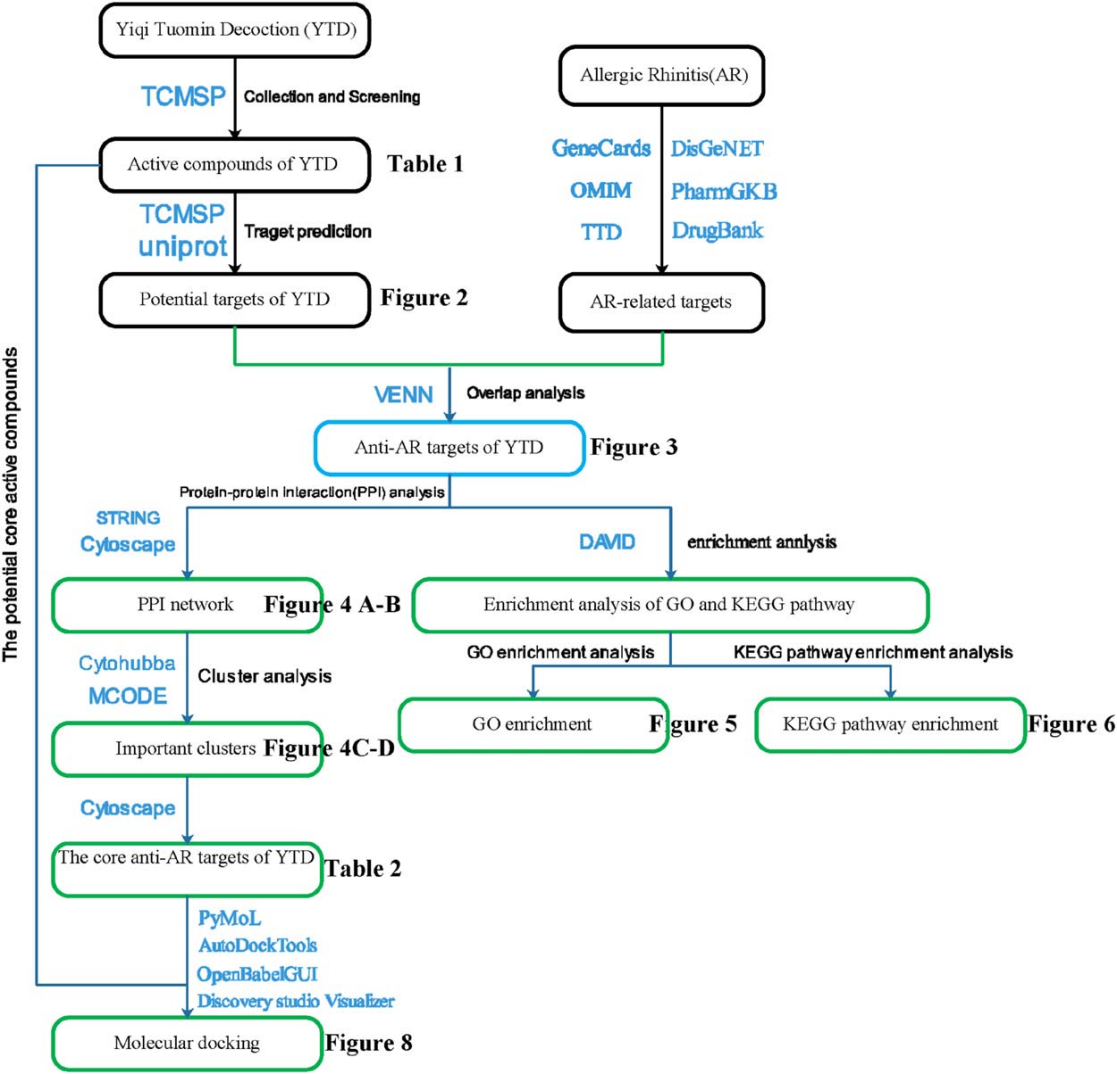
Workflow diagram of the network pharmacology-based study.

In our study, we provided information on predicting the bioactive ingredients, common targets, and signaling pathways of YTD against AR using network pharmacology and molecular docking methods. Figure 1 displays the workflow of our study. Our study results may provide effective theoretical support and expand the underlying mechanism of YTD against AR.

## Materials and methods

### Screening the active ingredients and targets of YTD and constructing the compound–target network

Traditional Chinese Medicine Systems Pharmacology (TCMSP) databases (https://tcmsp-e.com/tcmsp.php)^14^ were used to screen the compounds and the corresponding targets of *Hedysarum Multijugum Maxim* (HMM), *Saposhnikoviae Radix* (SR), *Paeoniae Radix Alba* (PRA), *Radix Bupleuri* (RB), *Cinnamomi Ramulus* (CR), *Xanthii Fructus* (XF), *Magnoliae Flos* (MaF), *A. Dahurica (Fisch.) Benth. Et Hook* (ADBEH), *Angelicae Sinensis Radix* (ASR), *Mume Fructus* (MF), and *Licorice* (Li) of YTD. In the TCMSP database, we could capture information related to herbal ingredients and drug-target by entering Chinese herbs^15^. Oral bioavailability (OB)^16^ and Drug-likeness (DL)^17^ were used as a method of evaluation to screen active molecules of Chinese herbs which were or not suitable for future research. Filter indicators of OB ≥30% and DL ≥0.18 were set to retrieve pharmacokinetic data from the TCMSP database. After that, the main ingredients and target proteins of YTD were obtained. Subsequently, The Uniprot (http://www.uniprot.org/) database was used to unite the genes of proteins, in which the reviewed and human ones were picked to filter by. Ultimately, the active ingredients of YTD and its target proteins were identified.

### Identification of targets for AR

The keyword ‘allergic rhinitis’, was entered in the GeneCards (https://www.genecards.org/), the OMIM database (http://www.omim.org/), TTD (http://db.idrblab.net/ttd/), DisGeNET (http://www.disgenet.org/) database, PharmGKB (https://www.pharmgkb.org/) database and DrugBank database (https://www. drugbank.ca/) to collect disease-related targets. By combining and deleting duplicates, the screened targets were the objective targets of AR for the next research.

### Construction of PPI network

Proteins often act in conjunction with other molecules^18^. The Venn database (http://bioinformatics.psb.ugent.be/webtools/Venn/) could be used to obtain the overlapped targets of active ingredients and diseases. The common targets of YTD and AR could be potential targets for YTD against AR. Then, we imported the related targets of AR into the STRING (http://stringdb.org/) database to construct the PPI network with the Multiple Proteins tool, in which we limited the *Homo sapiens* organism with the required score of highest confidence (0.900)^19^. Subsequently, the core targets network was visualized by using Cytoscape 3.9.0. Topographical values for the three parameters, which referred to betweenness centrality (BC), closeness centrality (CC), and degree centrality (DC), calculated by Cytoscape indicated the significance of relevant nodes in the network^20^. And then, the network parameters were systematically analyzed^21^. The corresponding target nodes with higher topographical values would be selected in the PPI network.

### Construction of the active ingredient–target network

Active ingredients and matched targets of YTD were imported into Cytoscape 3.9.0^22^ for visualization. The network of ingredient–target represented the relationship between them.

### GO and KEGG pathway enrichment analysis

The KOBAS-I database (http://kobas.cbi.pku.edu.cn/)^23^ was performed to analyze the enrichment of GO^24^. The enrichment of the KEGG pathway was analyzed by Sangerbox^25^.In the above respective databases, the target organism was set as *H. sapiens*, with false discovery rate (FDR) <0.05 and *P*<0.05 judged as cutoff values.

### Molecular docking

Molecular docking is a theoretical simulation method that is used to investigate the interaction between receptors and ligands and to predict their binding modes and affinity^26^. Therefore, classical molecular dynamics in PyMoL-2.2.0, AutoDockTools-1.5.7^6^, OpenBabelGUI-2.4.1, and Discovery studio Visualizer-v4.5.0 were utilized to analyze binding modes and affinity among the picked active ingredients of YTD and predicted targets. The crystal structures of the predicted proteins were downloaded from the RCSB Protein Data Bank (PDB, http://www.pdb.org/)^27^. The structures of picked active ingredients were downloaded from TCMSP. The procedure of hydrogenating, dehydrating, constructing the docking grid box, spilling the ligands from the receptors, and molecular docking and free binding energies calculation of putative targets and active ingredients were determined using AutoDockTools-1.5.7. The PyMOL-2.2.0 and Discovery Studio Visualizer-v4.5.0 were used for the visual analysis of the interaction among the active ingredients and predicted targets.

## Results

### Construction of compound–target network

**Figure 2:**
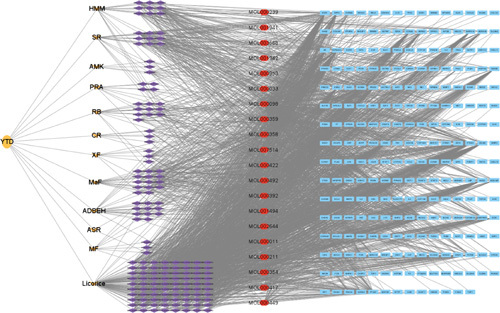
The compound–target network of *Yiqi Tuomin* Decoction (YTD) was screened by Cytoscape. Orange ellipses represent YTD, orange Vs represent the herbs, purple diamonds represent compounds, red hexagons represent shared compounds, and blue squares nodes represent the predicted targets. The gray edges indicate the interactions between the targets and the ingredients. ADBEH, *A. Dahurica (Fisch.) Benth. Et Hook*, ASR, *Angelicae Sinensis Radix*, CR, *Cinnamomi Ramulus*; HMM, *Hedysarum Multijugum Maxim*; Li, *Licorice*; MaF, *Magnoliae Flos*; MF, *Mume Fructus*; PRA, *Paeoniae Radix Alba*; RB, *Radix Bupleuri*; SR, *Saposhnikoviae Radix*; XF, *Xanthii Fructus*.

**Figure 3:**
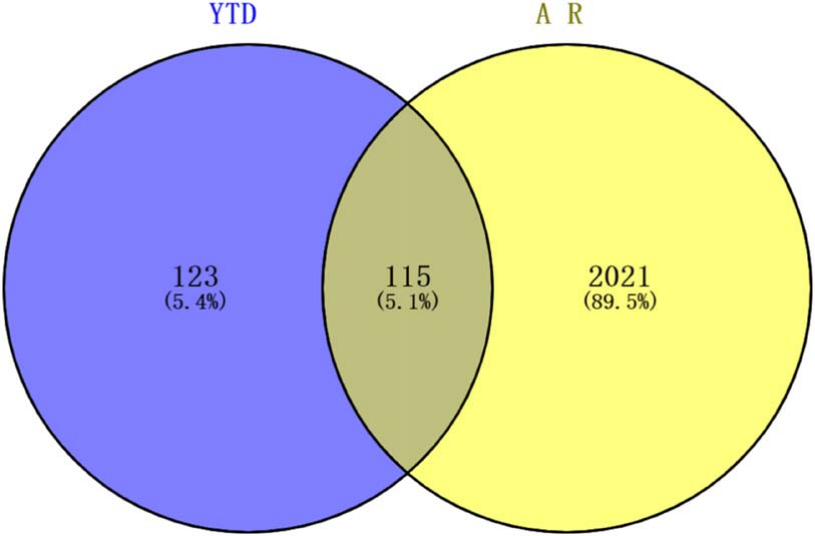
The diagram of the overlap of targets among *Yiqi Tuomin* Decoction (YTD) and allergic rhinitis (AR) was acquired by the Venn database.

In our study, there were 12 herbs in YTD. Based on the TCMSP database, a total of 169 active ingredients of YTD were identified after removing duplicates. These were listed in Supplementary 1, Supplemental Digital Content 1, http://links.lww.com/MS9/A135. As shown in Table 1, there were 20 active ingredients in YTD that were shared by two or more herbs. This revealed that different herbs in one formula could share the same or similar ingredients and targets, producing synergistic effects. The 238 targets of YTD were screened by TCMSP database and standardized gene names by UniProt and GeneCards. The preventive and therapeutic functions of Chinese herb formulas depended on the synergies of ingredient–target pathway^28^. Therefore, we identified effective targets for bioactive ingredients through a ligand-based prediction strategy and constructed a compound–target network from a holistic view using Cytoscape, as shown in Figure 2. The whole network comprised 420 nodes and 2523 edges altogether (Supplementary 2, Supplemental Digital Content 2, http://links.lww.com/MS9/A136). Based on the topological analysis, we determined the degree value of the node to be the primary screening criterion. In the network, the average degree value of bioactive ingredients was 14.86. With the degree value setting as ≥29.72 (twice the median degree), 12 candidate ingredients were identified, which showed that the demonstrated synergistic played a role in different herbs and ingredients. Meaningfully, we discovered that MOL000098 (quercetin), MOL000422 (kaempferol), MOL000173 (wogonin), MOL000378 (7-O-methylisomucronulatol), MOL003896 (7-methoxy-2-methyl isoflavone), MOL000392 (formononetin), MOL004328 (naringenin), MOL000354 (isorhamnetin), MOL000358 (beta-sitosterol), and MOL000497 (licochalcone A) interacted, respectively, with 144, 58, 43, 41, 39, 35, 35, 32, 31, and 31 targets, which became the key active ingredients in YTD. Polygenic predisposition is one of the major characteristics of AR. Studies related to the interaction between genes and the environment are helpful in revealing the pathogenesis of AR. AR-related targets were searched from GeneCards, TTD, DisGeNet, OMIM, PharmGKB, and Drugbank databases, resulting in a total of 2136 targets. As shown in Figure 3, YTD shared 115 common targets with AR, which were predicted to be the core targets for detecting the anti-AR activity of YTD.

**Table 1:** Top 20 compounds information of *Yiqi Tuomin* Decoction (YTD) network.

MOL_ID	Molecule_Name	Shared herbs
MOL000358	Beta-sitosterol	PRA, AMK, XF, ASR, SR, CR, MF
MOL000422	Kaempferol	PRA, RB, Li, HMM, MF
MOL000449	Stigmasterol	ADBEH, XF, RB, ASR, MF
MOL000359	Sitosterol	PRA, XF, SR, Li, CR
MOL000098	Quercetin	RB, Li, HMM, MF
MOL000211	Mairin	PRA, Li, HMM
MOL000354	Isorhamnetin	RB, Li, HMM
MOL001494	Mandenol	ADBEH, SR, MaF
MOL000417	Calycosin	Li, HMM
MOL000239	Jaranol	Li, HMM
MOL000392	Formononetin	Li, HMM
MOL000033	(3S,8S,9S,10R,13R,14S,17R)-10,13-dimethyl-17-[(2R,5S)-5-propan-2-yloctan-2-yl]-2,3,4,7,8,9,11,12,14,15,16,17-dodecahydro-1H-cyclopenta[a]phenanthren-3-ol	AMK, HMM
MOL000953	CLR	ADBEH, MF
MOL000492	(+)-catechin	PRA, CR
MOL001941	Ammidin	ADBEH, SR
MOL007514	Methyl icosa-11,14-dienoate	ADBEH, SR
MOL002644	Phellopterin	ADBEH, SR
MOL001942	Isoimperatorin	ADBEH, SR
MOL003588	Prangenidin	ADBEH, SR
MOL000011	(2R,3R)-3-(4-hydroxy-3-methoxy-phenyl)-5-methoxy-2-methylol-2,3-dihydropyrano[5,6-h][1,4]benzodioxin-9-one	XF, SR

ADBEH, *A. Dahurica (Fisch.) Benth. Et Hook*, ASR, *Angelicae Sinensis Radix*, CR, *Cinnamomi Ramulus*; HMM, *Hedysarum Multijugum Maxim*; Li, *Licorice*; MaF, *Magnoliae Flos*; MF, *Mume Fructus*; PRA, *Paeoniae Radix Alba*; RB, *Radix Bupleuri*; SR, *Saposhnikoviae Radix*; XF, *Xanthii Fructus*.

### PPI network of shared targets

To further investigate the core pharmacological underlying mechanisms of YTD against AR, a topological approach was employed to assess the core network.

**Figure 4:**
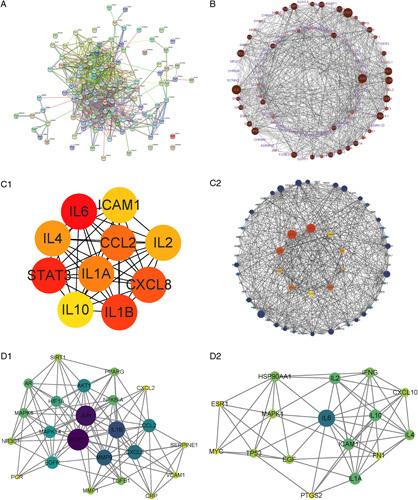
(A) Protein–Protein interaction (PPI) network of common targets exported by STRING database (high confidence ≥0.700, medium≥5%); (B) The reconstructed PPI network was screened by Cytoscape and analyzed by Network Analyzer. (C) The hub genes were screened from the PPI network using the CytoHubba plugin. The node color is from pale yellow to red (C_1); C_2 shows the hub gene neighbors. The inner circle represents the hub gene, the outer circle represents the hub gene neighbors, both corresponding degrees gradually larger. (D) PPI network based on clustery analysis using the MCODE plugin. (D_1) Cluster 1: 23 nodes, 113 edges, score: 10.273; (D_2) Cluster 2: 16 nodes, 61 edges, score: 8.133.

PPI network is recognized as a good way to investigate the effect of multiple proteins in gene-related diseases. Consequently, the PPI network of shared targets was constructed from the STRING database (Fig. 4A). For better visualization, the original data from the STRING database was input into Cytoscape. The results of the reconstruction of the PPI network showed that the whole network comprised 106 nodes (9 disconnected nodes have been deleted) and 749 edges (Fig. 4B). In the reconstructed network, the node size was proportional to the target degree value.

To illuminate the biological functions of shared targets, we classified the central PPI network into two clusters using two Cytoscape plugins, Cytohubba and MCODE. We selected the top 10 genes ranked by MCC (Maximal Clique Centrality) using the Cytohubba plugin, and also screened the top 10 genes with interaction neighbors (Fig. 4C). As shown in Figure 4D, we implemented cluster analysis using the MCODE plugin to produce a highly connected sub-network, and divided the correlated nodes into two groups.

### Enrichment analysis of the GO and KEGG pathways

#### GO enrichment

**Figure 5:**
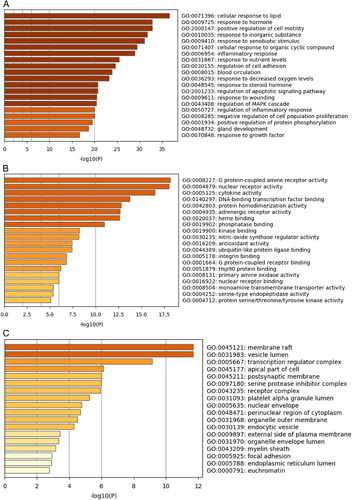
Gene Ontology (GO) enrichment analysis of the common targets was employed by the KOBAS-I, and the results of the analysis were colored by *P* values. (A) Biological processes, (B) molecular function, and (C) cellular ingredients.

GO enrichment analysis was performed on the 115 predicted targets to indicate the various mechanisms of YTD against AR, including biological processes (BP), molecular function (MF), and cellular ingredient (CC). The top 20 GO-enriched terms were determined (Fig. 5). Through GO terms data, we discovered that response to hormone (steroid hormone), inflammatory response, blood circulation, oxygen levels, nutrient levels, nitric-oxide synthase regulator activity, G protein-coupled amine receptor activity, transcription factor binding, and Hsp90 protein binding were enriched in the targets. Meanwhile, the results indicated that a genetic perspective with multiple synergies may be an effective research direction for YTD against AR.

#### KEGG

**Figure 6:**
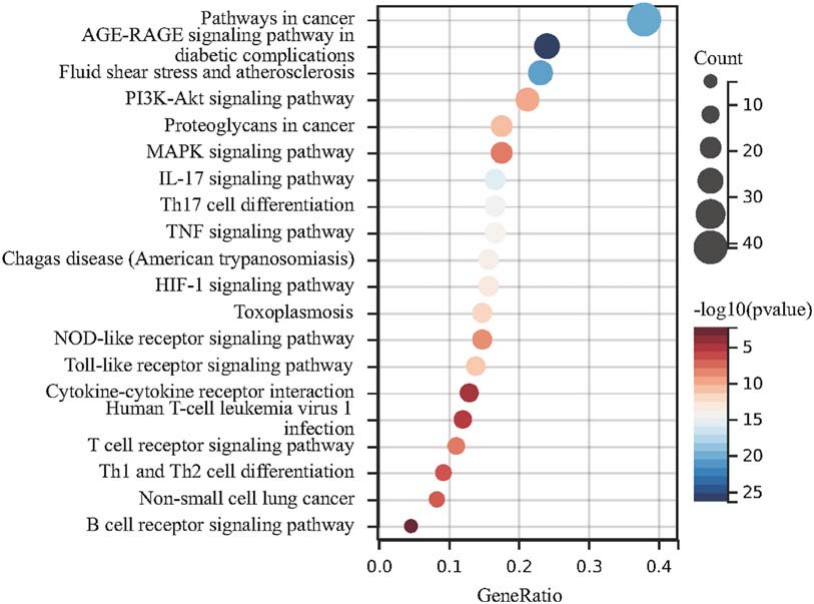
Kyoto Encyclopedia of Genes and Genomes (KEGG) pathway enrichment of the common targets is employed by Sangerbox 3.0. Each bubble delegates an enriched function, and the size of the bubble is proportional to the count. Each bubble represents different clusters.

KEGG pathway enrichment analysis has been carried out for 115 predicted targets. All genes in the genome were used as the enrichment background. The key 20 enriched KEGG pathways were determined and shown in Figure 6 and Table 2. The whole enrichment analysis results of the KEGG pathway were determined in Supplementary 3, Supplemental Digital Content 3, http://links.lww.com/MS9/A137. Based on the annotation of the KEGG pathway, we believed that the core signaling pathways, such as MAPK signaling pathway, Th17 cell differentiation, IL-17 signaling pathway, and Th1 and Th2 cell differentiation, might be the key pharmacological mechanism of YTD for AR.

**Table 2:** KEGG enrichment analysis results of common genes.

Term	ID	Count	Background number	*P*	Corrected *P*	Genes
Pathways in cancer	hsa05200	40	530	3.65E−44	8.75E−42	*BCL2L1*, *TGFB1*, *MYC*, *PTGS2*, *NFKBIA*, *EGFR*, *HIF1A*, *AKT1*, *EGF*, *STAT3*, *STAT1*, *NFE2L2*, *JUN*, *GSTP1*, *PTGER3*, *PRKCA*, *MAPK1*, *CXCL8*, *MAPK8*, *ERBB2*, *NOS2*, *IFNG*, *RXRB*, *PPARG*, *VEGFA*, *ESR2*, *RELA*, *TP53*, *FN1*, *ESR1*, *GSTM1*, *HMOX1*, *IL6*, *IL4*, *IL2*, *MMP9*, *HSP90AA1*, *BCL2*, *MMP2*, *MMP1*
AGE–RAGE signaling pathway in diabetic complications	hsa04933	25	100	2.75E−39	3.30E−37	*CCL2*, *TGFB1*, *MAPK14*, *AKT1*, *IL1A*, *IL1B*, *SERPINE1*, *STAT3*, *STAT1*, *JUN*, *MAPK1*, *ICAM1*, *MAPK8*, *NOS3*, *PRKCA*, *PRKCD*, *VEGFA*, *VCAM1*, *RELA*, *FN1*, *SELE*, *IL6*, *CXCL8*, *MMP2*, *BCL2*
Fluid shear stress and atherosclerosis	hsa05418	25	139	4.28E−36	3.42E−34	*CCL2*, *MAPK14*, *AKT1*, *IL1A*, *IL1B*, *NFE2L2*, *JUN*, *ICAM1*, *MAPK8*, *KDR*, *NOS3*, *IFNG*, *MMP9*, *PLAT*, *VEGFA*, *VCAM1*, *RELA*, *TP53*, *SELE*, *HMOX1*, *GSTP1*, *GSTM1*, *HSP90AA1*, *MMP2*, *BCL2*
PI3K–Akt signaling pathway	hsa04151	22	354	1.98E−22	4.75E−21	*BCL2L1*, *EGFR*, *AKT1*, *EGF*, *MAPK1*, *MYC*, *KDR*, *ERBB2*, *ERBB3*, *NOS3*, *PRKCA*, *CHRM2*, *CHRM1*, *VEGFA*, *RELA*, *TP53*, *FN1*, *IL6*, *IL4*, *IL2*, *HSP90AA1*, *BCL2*
Proteoglycans in cancer	hsa05205	19	203	1.29E−22	3.45E−21	*ERBB2*, *TGFB1*, *STAT3*, *FN1*, *ERBB3*, *PRKCA*, *ESR1*, *EGFR*, *HIF1A*, *MAPK14*, *AKT1*, *MAPK1*, *VEGFA*, *PLAU*, *MMP9*, *TP53*, *MYC*, *MMP2*, *KDR*
MAPK signaling pathway	hsa04010	19	295	9.61E−20	1.36E−18	*ERBB2*, *TGFB1*, *MYC*, *TP53*, *ERBB3*, *PRKCA*, *EGFR*, *MAPK8*, *JUN*, *EGF*, *MAPK14*, *AKT1*, *CD14*, *MAPK1*, *VEGFA*, *IL1A*, *IL1B*, *RELA*, *KDR*
Metabolic pathways	hsa01100	19	1433	4.40E−08	1.07E−07	*PTGS2*, *NOS2*, *NOS3*, *ACACA*, *CYP1A1*, *GSTM1*, *ALOX5*, *HMOX1*, *PTGS1*, *CYP1A2*, *CAT*, *MAOB*, *MAOA*, *AR*, *PLB1*, *LTA4H*, *CYP3A4*, *AKR1B1*, *GSTP1*
IL-17 signaling pathway	hsa04657	18	93	1.14E−26	6.83E−25	*CCL2*, *PTGS2*, *CXCL2*, *NFKBIA*, *IFNG*, *CXCL8*, *MAPK8*, *JUN*, *HSP90AA1*, *MAPK14*, *IL6*, *IL4*, *MAPK1*, *MMP9*, *CXCL10*, *IL1B*, *RELA*, *MMP1*
Th17 cell differentiation	hsa04659	18	107	1.11E−25	5.32E−24	*TGFB1*, *NFKBIA*, *STAT3*, *STAT1*, *IFNG*, *MAPK8*, *JUN*, *RXRB*, *AHR*, *IL6*, *IL4*, *MAPK1*, *IL2*, *HIF1A*, *HSP90AA1*, *IL1B*, *RELA*, *MAPK14*
TNF signaling pathway	hsa04668	18	112	2.34E−25	9.35E−24	*CCL2*, *PTGS2*, *IRF1*, *CXCL2*, *NFKBIA*, *SELE*, *MAPK8*, *JUN*, *MAPK14*, *AKT1*, *MAPK1*, *VCAM1*, *ICAM1*, *MMP9*, *CXCL10*, *IL1B*, *RELA*, *IL6*
Chagas disease (American trypanosomiasis)	hsa05142	17	103	3.55E−24	1.22E−22	*CCL2*, *TGFB1*, *NOS2*, *NFKBIA*, *AKT1*, *IL10*, *CXCL8*, *MAPK8*, *JUN*, *SERPINE1*, *MAPK14*, *IFNG*, *MAPK1*, *IL2*, *IL1B*, *RELA*, *IL6*
HIF-1 signaling pathway	hsa04066	17	109	8.52E−24	2.56E−22	*ERBB2*, *NOS2*, *NOS3*, *STAT3*, *SERPINE1*, *IFNG*, *EGFR*, *HMOX1*, *HIF1A*, *PRKCA*, *IL6*, *AKT1*, *MAPK1*, *VEGFA*, *RELA*, *EGF*, *BCL2*
Toxoplasmosis	hsa05145	16	113	7.72E−22	1.69E−20	*BCL2L1*, *TGFB1*, *NOS2*, *STAT3*, *STAT1*, *AKT1*, *IFNG*, *ALOX5*, *NFKBIA*, *MAPK14*, *IL10*, *MAPK1*, *CD40LG*, *RELA*, *MAPK8*, *BCL2*
NOD-like receptor signaling pathway	hsa04621	16	178	6.33E−19	6.85E−18	*BCL2L1*, *CCL2*, *CXCL2*, *NFKBIA*, *STAT1*, *CXCL8*, *MAPK8*, *JUN*, *PRKCD*, *MAPK14*, *IL6*, *MAPK1*, *HSP90AA1*, *IL1B*, *RELA*, *BCL2*
Cytokine-cytokine receptor interaction	hsa04060	14	294	4.21E−13	1.84E−12	*CCL2*, *TGFB1*, *CXCL2*, *CD40LG*, *IL10*, *CXCL8*, *IL6*, *IFNG*, *IL4*, *IL2*, *IL1A*, *CXCL11*, *CXCL10*, *IL1B*
Human T cell leukemia virus 1 infection	hsa05166	13	219	2.12E−13	1.00E−12	*BCL2L1*, *TGFB1*, *MYC*, *TP53*, *NFKBIA*, *JUN*, *IL6*, *AKT1*, *MAPK1*, *IL2*, *ICAM1*, *RELA*, *MAPK8*
T cell receptor signaling pathway	hsa04660	12	103	1.03E−15	6.02E−15	*CD40LG*, *AKT1*, *IL10*, *NFKBIA*, *JUN*, *MAPK14*, *IFNG*, *IL4*, *MAPK1*, *IL2*, *RELA*, *MAPK8*
Th1 and Th2 cell differentiation	hsa04658	10	92	5.17E−13	2.22E−12	*STAT1*, *IFNG*, *NFKBIA*, *JUN*, *MAPK14*, *IL4*, *MAPK1*, *IL2*, *MAPK8*, *RELA*
Non-small cell lung cancer	hsa05223	9	66	1.21E−12	4.90E−12	*ERBB2*, *STAT3*, *PRKCA*, *EGFR*, *RXRB*, *AKT1*, *MAPK1*, *TP53*, *EGF*
B cell receptor signaling pathway	hsa04662	5	82	5.95E−06	1.11E−05	*AKT1*, *RELA*, *NFKBIA*, *JUN*, *MAPK1*

### Molecular docking

**Figure 7:**
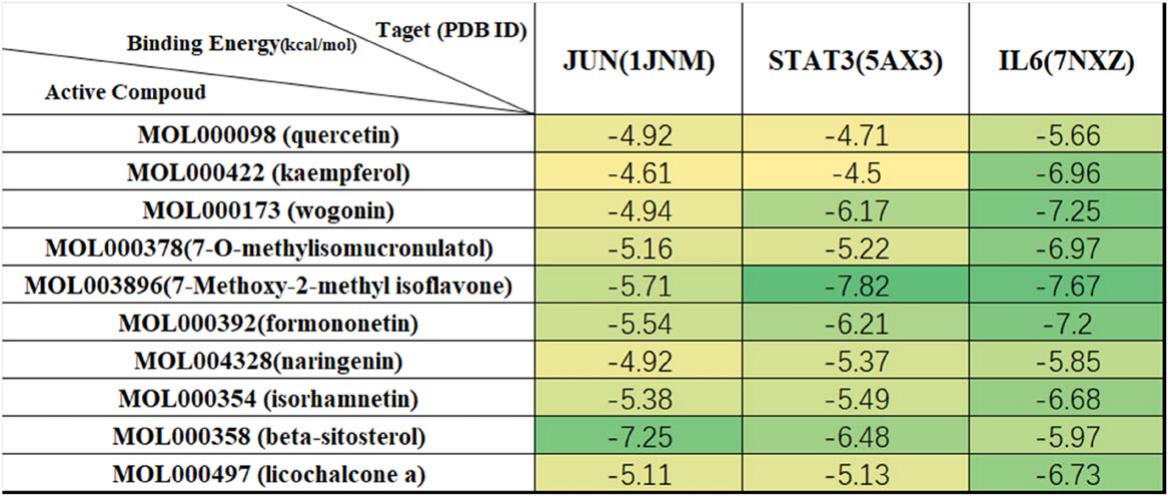
The binding results of compound and core targets.

**Figure 8:**
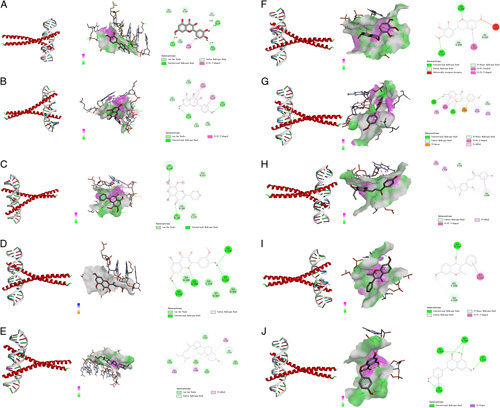
The results of molecular docking of the main active ingredients of *Yiqi Tuomin* Decoction (YTD) were determined using AutoDockTools-1.5.7. The PyMOL-2.2.0 and Discovery Studio Visualizer-v4.5.0 were employed to visualize and analyze the interaction and binding mode of the active ingredients. (A) Quercetin–JUN; (B) Kaempferol–JUN; (C) Wogonin–JUN; (D) Isorhamnetin–JUN; (E) Beta-sitosterol–JUN; (F) 7-O-methylisomucronulatol–JUN; (G) Formononetin–JUN; (H) Licochalcone A–JUN; (I) 7-Methoxy-2-methyl isoflavone–JUN; (J) Naringenin–JUN.

**Figure 9:**
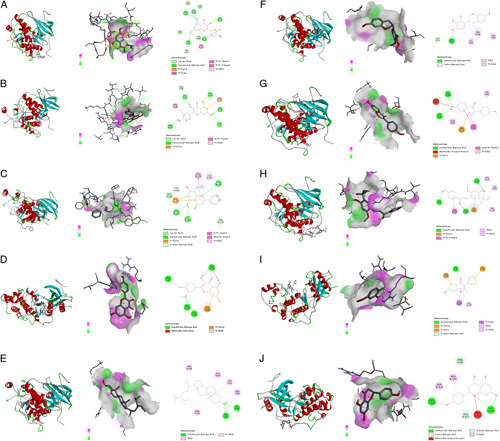
The results of molecular docking of the main active ingredients of *Yiqi Tuomin* Decoction (YTD) were determined using AutoDockTools-1.5.7. The PyMOL-2.2.0 and Discovery Studio Visualizer-v4.5.0 were employed to visualize and analyze the interaction and binding mode of the active ingredients. (A) Quercetin–STAT3; (B) Kaempferol–STAT3; (C) Wogonin–STAT3; (D) Isorhamnetin–STAT3; (E) Beta-sitosterol–STAT3; (F) 7-O-methylisomucronulatol–STAT3; (G) Formononetin–STAT3; (H) Licochalcone A–STAT3; (I) 7-Methoxy-2-methyl isoflavone–STAT3; (J) Naringenin–STAT3.

**Figure 10:**
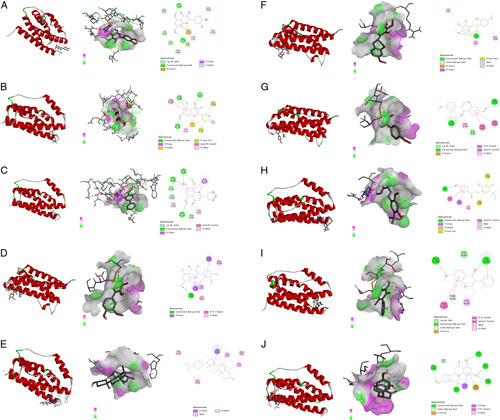
The results of molecular docking of the main active ingredients of *Yiqi Tuomin* Decoction (YTD) were determined using AutoDockTools-1.5.7. The PyMOL-2.2.0 and Discovery Studio Visualizer-v4.5.0 were employed to visualize and analyze the interaction and binding mode of the active ingredients. (A) Quercetin–IL6; (B) Kaempferol–IL6; (C) Wogonin–IL6; (D) Isorhamnetin–IL6; (E) Beta-sitosterol–IL6; (F) 7-O-methylisomucronulatol–IL6; (G) Formononetin–IL6; (H) Licochalcone A–IL6; (I) 7-Methoxy-2-methyl isoflavone–IL6; (J) Naringenin–IL6.

According to the network pharmacology results, the top 10 ingredients were screened. These were MOL000098 (quercetin), MOL000422 (kaempferol), MOL000173 (wogonin), MOL000378 (7-O-methylisomucronulatol), MOL003896 (7-methoxy-2-methyl isoflavone), MOL000392 (formononetin), MOL004328 (naringenin), MOL000354 (isorhamnetin), MOL000358 (beta-sitosterol), and MOL000497 (licochalcone A). Then molecular docking was implemented gradually with JUN (PDB ID:1JNM), STAT3 (PDB ID:5AX3), and IL6 (PDB ID:7NXZ), which were the top three target proteins. The binding energy of ingredients and core proteins is shown in Figure 7. Figures 8–10 depicted visual images of the most stable complex between ligands and receptors.

Figure 8 displayed the docking results that JUN could interact with quercetin, kaempferol, wogonin, isorhamnetin, beta-sitosterol, 7-O-methylisomucronulatol, formononetin, licochalcone A, 7-methoxy-2-methyl isoflavone, and naringenin.

Figure 8A showed that quercetin forms three conventional hydrogen bonds with DT-207, DA-209, and DA-314 and one carbon–hydrogen bond with DT-315. Figure 8B showed that kaempferol formed two conventional hydrogen bonds with DA-318 and DG-320 and the interaction of the Pi–Pi T-shaped with DC-204 and DC-319. Figure 8C showed that wogonin formed two conventional hydrogen bonds with DC-310 and DG-308. Figure 8D showed that isorhamnetin formed four conventional hydrogen bonds with DC-310, DG-308, DT-215, and DC-216, and one carbon–hydrogen bond with DC-216. Figure 8E showed that beta-sitosterol formed one carbon–hydrogen bond with DC-216, and the interaction of the Pi–Pi T-shaped with DA-309, DG-308, and DA-306. Figure 8F showed that 7-O-methylisomucronulatol formed two carbon–hydrogen bonds with DA-214 and DA-309, and two Pi-donor hydrogen bonds with DG-308 and DC-310, and the interaction of the Pi–Pi T-shaped with DA-309 and DG-308. Figure 8G showed that formononetin formed two conventional hydrogen bonds with SER-269 and LYS-273 and one carbon–hydrogen bond with DC-310, and the interaction of the Pi–Pi T-shaped with DG-308. Figure 8H showed that licochalcone A formed two carbon–hydrogen bonds with DC-316 and DG-208, and the interaction of the Pi–Pi T-shaped with DG-208. Figure 8I showed that 7-methoxy-2-methyl isoflavone formed three carbon–hydrogen bonds with DG-208 and DA-209, and the interaction of the Pi–Pi T-shaped with DT-315. Figure 8J showed that naringenin formed four conventional hydrogen bonds with DC-310, DA-309, DG-308, and DT-215.

Figure 9 displayed the docking results that STAT3 could interact with quercetin, kaempferol, wogonin, isorhamnetin, beta-sitosterol, 7-O-methylisomucronulatol, formononetin, licochalcone A, 7-methoxy-2-methyl isoflavone, and naringenin. Figure 9A showed that quercetin forms five conventional hydrogen bonds with ARG-126, TYR-122, TYR-119, LYS-351, and LYS-352, and the interaction of the Pi–Pi T-shaped with TYR-353. Figure 9B showed that kaempferol formed five conventional hydrogen bonds with ARG-343, LEU-67, PHE-69, ASN-78, and GLY-76, and the interaction of the Pi–Pi T-shaped with PHE-344. Figure 9C shows that wogonin formed three conventional hydrogen bonds with ARG-185, ILE-189, and THR-176, and the interaction of the Pi–Pi T-shaped with LEU-175. Figure 9D showed that isorhamnetin formed five conventional hydrogen bonds with ARG-58, PHE-174, LEU-175, and ASP-326. Figure 9E showed that beta-sitosterol formed three conventional hydrogen bonds with ASP-91 and LYS-90. Figure 9F showed that 7-O-methylisomucronulatol formed one conventional hydrogen bond with ARG-15, and one carbon–hydrogen bond with ASP-79. Figure 9G showed that formononetin formed two conventional hydrogen bonds with ASP-91 and LYS-90. Figure 9H showed that licochalcone A formed three conventional hydrogen bonds with VAL-350, GLN-305, and TYR-306. Figure 9I showed that 7-methoxy-2-methyl isoflavone formed one conventional hydrogen bond with ARG-185. Figure 9J showed that naringenin formed two conventional hydrogen bonds with GLU-316 and LYS-320, and one carbon–hydrogen bond with ARG-68, and one Pi-Donor hydrogen bond with ALA-317.

Figure 10 displayed the docking results that IL6 could interact with quercetin, kaempferol, wogonin, isorhamnetin, beta-sitosterol, 7-O-methylisomucronulatol, formononetin, licochalcone A, 7-methoxy-2-methyl isoflavone, and naringenin.

Figure 10A showed that quercetin formed two conventional hydrogen bonds with CYS-49 and ASP-159. Figure 10B shows that kaempferol formed four conventional hydrogen bonds with THR-42, ASP-159, HIS-163, and NLE-48. Figure 10C shows that wogonin formed four conventional hydrogen bonds with ARG-39, ASP-159, and THR-42. Figure 10D showed that isorhamnetin formed one conventional hydrogen bond with ASP-159, and the interaction of the Pi–Pi T-shaped with HIS-163 and NLE-48. Figure 10E showed that the interaction between beta-sitosterol and IL6 could not depend on hydrogen bonds. Figure 10F showed that 7-O-methylisomucronulatol formed one conventional hydrogen bond with HIS-163 and one carbon–hydrogen bond with ARG-39. Figure 10G showed that formononetin formed two conventional hydrogen bonds with HIS-163 and ASP-159 and the interaction of the Pi–Pi T-shaped with THR-42 and THR-162. Figure 10H showed that licochalcone A formed two conventional hydrogen bonds with ARG-39 and NLE-48. Figure 10I showed that 7-methoxy-2-methyl isoflavone formed two conventional hydrogen bonds with ARG-103 and HIS-163, and one carbon–hydrogen bond with THR-42. Figure 10J showed that naringenin formed five conventional hydrogen bonds with GLU-68, GLN-74, GLN-182, CYS-72, and SER-175, and one carbon–hydrogen bond with SER-75.

The stability of conformation between receptor and ligand is proportional to the binding energy and the affinity. As Figure 7 shown, the free docking results showed that the binding energies were between −7.82 and −4.50 kcal/mol, which implied stable binding. The free binding energy of JUN to beta-sitosterol was −7.25 kcal/mol, the free binding energy of STAT3 to 7-methoxy-2-methyl isoflavone was −7.82 kcal/mol and the free binding energy of IL6 to wogonin, 7-methoxy-2-methyl isoflavone, and formononetin were −7.25 kcal/mol, −7.67 kcal/mol, and −7.2 kcal/mol, respectively.

The above results indicate that, among the active ingredients, the proton and oxygen of the hydroxyl groups, as well as the ketone oxygen, tended to form hydrogen bonds with the active site residues of proteins.

We found in our study that beta-sitosterol revealed better binding activity with JUN, 7-methoxy-2-methyl isoflavone revealed better binding activity with STAT3, and wogonin, 7-methoxy-2-methyl isoflavone, and formononetin revealed better binding activity with IL6. Therefore, we identified these herbal active ingredients as drug-candidate molecules. Molecular docking technology provided a strategy for evaluating the binding mode between the herbal ingredients and related targets. However, the results indicate that reliable experiments still need to be performed to validate the herbal active ingredients.

## Discussion

AR is recognized as an abnormal inflammation of the membrane lining the nose^29^. It is characterized by symptoms of sneezing, itching, rhinorrhea, nasal congestion, and nasal hypersensitivity^30^. It is driven by type 2 cells of mucosal inflammation and caused by IgE-mediated reactions^31^. The complexity of the pathogenesis of AR and the multiple pathways involved provide many targets for drug treatment. However, to date, no single drug has been reported to be totally effective. For patients, AR is not only a physical trauma but also an economic burden. It disturbs sleep quality, reduces working passion, weakens cognitive functions, and affects the quality of life and irritability.

TCM herbal formulas can help improve the treatment of chronic diseases through the ingredient–target-effect mechanism and the concept of syndrome differentiation and treatment. YTD is a clinical treatment method for allergic reactions and inflammation induced by hypersensitivity. However, the specific pathophysiological and pharmacologic mechanisms of YTD against AR have not yet been fully understood. In our study, to prove the underlying mechanisms of the curative effects of YTD against AR, we focused on two aspects: pharmacology and pathophysiology. In terms of pharmacology, we screened the active ingredients and identified core targets that could play crucial regulatory roles in treatment and then identified key enriched terms and biological pathways^15^. In terms of pathophysiology, we understood the pathogenesis of AR and provided an approach to treat AR by combining pharmacology.

A network pharmacology approach, which is related to the analysis of network models and systems biology^32^, combines traditional pharmacology with bioinformatics, cheminformatics, and network biology. The network pharmacology method analyzes complex network relationships concerning the multi-ingredient, multi-target, and multi-channel aspects, similar to the theory of TCM based on syndrome differentiation^33^.

The purpose of the study was to explore the underlying mechanism of YTD against AR using the methods of network pharmacology and molecular docking. From the TCMSP database, 169 chemical ingredients were screened. Among these chemical ingredients, quercetin, kaempferol, and wogonin were found to act on more targets compared to other active ingredients in the network.

We collected AR-related targets from OMIM, GeneCards, Drugbank, PharmGKB, TTD, and DisGeNet databases. From the above databases, YTD and AR have been found to have about 115 common targets. These targets were considered potential targets of YTD against AR. To predict the core targets of YTD against AR, a PPI network was constructed to express protein–protein interactions. The results indicated that JUN, STAT3, IL6, AKT1, IL1B, RELA, HSP90AA1, TP53, VEGFA, EGFR, and CXCL8, particularly JUN, STAT3, and IL6, may be the core targets.

GO and KEGG pathway enrichment analyses were conducted to predict the mechanism of YTD against AR. GO terms analysis results showed that the target genes were chiefly enriched in biological functions such as response to hormone (steroid hormone), inflammatory response, blood circulation, oxygen levels, nutrient levels, G protein-coupled amine receptor activity, nitric-oxide synthase regulator activity, transcription factor binding, and Hsp90 protein binding. KEGG pathway analysis results showed that YTD involved multiple signaling pathways in treating AR, with the key signaling pathways referring to the MAPK signaling pathway, IL-17 signaling pathway, Th17 cell differentiation, and Th1 and Th2 cell differentiation. The MAPK signaling pathway is one of the primary regulators of inflammatory response through transcriptional regulation of cytokines production. IL-17A plays a key role in the immune expression of IL-17, and sometimes Th17 cells could induce the production of IL-17. STAT3 (activator of transcription 3) and IL6 activate the IL-17-induced gene expression in the analysis of signal transduction^34^.

Some studies indicate that T helper (Th) cells play a vital role in the pathological process of AR^35^. The inflammatory pathway involved in AR is an IgE-mediated inflammatory response^36^. Interactions between allergens and nasal mucosa result in allergen-specific IgE binding to eosinophils, basophils, and mast cells. Meanwhile, the IgE-mediated mast cell response enhances the recruitment of T helper cells (Th1 and Th2), eosinophils, and basophils^37^. Th1 and Th2 cells could affect the production of IgE. Th1 cells secrete IFN‑γ and IL‑12, which inhibit the production of IgE^38,39^. Th2 cells secrete IL-13, IL‑4, and IL‑5, which provoke IgE production and induce an IgG isotype switch to IgG1^40^. Hsieh *et al*.^41^ indicated that Th2 cells played a modulated role in regulating downstream cell differentiation and proliferation, switching to secreting IgE, IgG1, and eosinophils. Normally, to maintain a normal immune status, Th1 and Th2 cells were relatively balanced in number.

The enriched results of GO and KEGG pathways indicate that YTD could play an anti-allergic effect by inhibiting inflammatory and allergy-related pathways. The results of network pharmacology show that the top 10 ingredients, which are MOL000098 (quercetin), MOL000422 (kaempferol), MOL000173 (wogonin), MOL000378 (7-O-methylisomucronulatol), MOL003896 (7-methoxy-2-methyl isoflavone), MOL000392 (formononetin), MOL004328 (naringenin), MOL000354 (isorhamnetin), MOL000358 (beta-sitosterol), and MOL000497 (licochalcone A) may be the core bioactive chemical ingredients in our studies. Quercetin which is a naturally occurring polyphenol flavonoid, is known for restraining histamine production, being anti-allergic and anti-inflammatory, regulating the Th1/Th2 stability^42^, and decreasing IgE antibody releasing by B cells^43^. Kaempferol is a flavonoid that has shown effective inhibitory factors on inflammation in the airways. It can inhibit the production and release of inflammatory cytokines and chemokines, as well as the stimulation of mast cells^44^. Lee *et al*.^45^ investigated the effects of kaempferol and quercetin on TNF-α and IL4 levels and found that they decreased. However, in sensitized human mast cells, kaempferol showed a better suppressing rate than quercetin. Wogonin is a type of polyphenol commonly found in plants that is used in the treatment of allergies and inflammation due to its toxin-removing and thermal-cleansing properties^46^. It plays an inhibitory effect on IgE production due to its antioxidative effect. The anti-inflammatory action of wogonin played a crucial role in anti-edema activity^47^. 7-O-methylisomucronulatol, 7-methoxy-2-methyl isoflavone, isorhamnetin, and formononetin are commonly found in the *Fabaceae* family^48^. They reduced the production of inflammatory cytokines by inhibiting the MAPK and NF-κB signaling pathways^49^. Naringenin, a flavanone flavonoid, has been reported to ameliorate inflammatory response by suppressing MAPK activation^50^. From molecular docking results, beta-sitosterol showed better binding activity with JUN, 7-methoxy-2-methyl isoflavone showed better binding activity with STAT3, and wogonin, 7-methoxy-2-methyl isoflavone, and formononetin showed better binding activity with IL6. This indicates that they may be potential pharmaceutical active ingredients of YTD against AR.

## Conclusions

Based on network pharmacology and the theory of syndrome differentiation and treatment, our study illustrated the features of YTD that are responsible for drug synergy and predicted the possible pharmacological mechanisms of YTD against AR.

In our study, we implemented the methods of network pharmacology and molecular docking technology to conduct a preliminary exploration of the active ingredients and potential targets of YTD against AR. Our results showed wogonin, 7-methoxy-2-methyl isoflavone, formononetin, and beta-sitosterol might be the core bioactive ingredients from YTD in the treatment of AR. Additionally, the targets JUN, STAT3, and IL6 might be potential therapeutic target proteins of YTD against AR. The results also suggested that the mechanism of YTD against AR may involve the bioactive ingredients acting on JUN, STATS, IL6, and other targets, while regulating the MAPK signaling pathway, IL-17 signaling pathway, Th17 cell differentiation, and Th1 and Th2 cell differentiation, thus exerting immunomodulatory and anti-inflammatory effects.

Our results showed that it not only provided new insights for a more comprehensive investigation of the chemical substance basis and pharmacology of YTD but also demonstrated a feasible method for potential drug discovery in herbal medicine.

## Ethical approval

None declared.

## Consent

None declared.

## Sources of funding

This study was financially supported by the Key Project of the Jiangsu Provincial Health Commission (ZD2021016) and the Construction program of the Jiangsu Provincial Administration of Traditional Chinese Medicine (No. 2021-7, Chen Xiaoning).

## Author contribution

W.Z.: conceptualization, methodology, formal analysis, data curation, and writing – original draft; Q.Z.: data curation and review and editing; X.C.: funding acquisition and review and editing; J.Z.: validation; J.S. and L.C.: funding acquisition and project administration.

## Conflicts of interest disclosure

The authors declare that they have no known competing financial interests or personal relationships that could have appeared to influence the work reported in this paper.

## Research registration unique identifying number (UIN)

1. Name of the registry: not applicable.
2. Unique identifying number or registration ID: not applicable.
3. Hyperlink to your specific registration (must be publicly accessible and will be checked): not applicable.

## Guarantor

Weixin Zhang.

## Provenance and peer review

Not commissioned, externally peer-reviewed.

## Supplementary Material
